# Combined Intestinal and Biliary Stenting in Gastric Outlet and Biliary Obstruction

**DOI:** 10.4021/gr2009.02.1273

**Published:** 2009-01-20

**Authors:** Guang Chuan Wang, Feng Liu, Tian Hua Xie, Fu Li Liu, Chun Qing Zhang

**Affiliations:** a Department of Gastroenterology, Provincial hospital affiliated to Shangdong University, Jinan, Shangdong, China, 250021

**Keywords:** Gastric outlet obstruction, Biliary obstruction, Stents, Percutaneous transhepatic cholangial drainage

## Abstract

**Background:**

Combined intestinal and biliary stenting is one of the effective palliative methods for patients with malignant gastric outlet and biliary obstruction. This study was to evaluate the effect of combined intestinal and biliary stenting in the palliation of gastric outlet and biliary obstruction.

**Methods:**

Thirty-two patients with malignant gastric outlet and biliary obstruction underwent combined intestinal and biliary stenting. Intestinal stents were implanted by means of endoscopy and X-ray guidance. The subsequent biliary stents were implanted by percutaneous transhepatic cholangial drainage. The biliary stent pass through the side hole of intestinal stent mesh and its distal segment was located in the lumen of intestinal stent.

**Results:**

Thirty-four intestinal stents and 32 biliary stents for 32 patients were implanted successfully. No lethal complications occurred. The average survival was 164 days.

**Conclusions:**

The combined intestinal and biliary stenting is an effective and safe method for palliation of gastric outlet and biliary obstructions. The short-term results are satisfactory.

## Introduction

Patients with malignant tumors in descending duodenum, hepatopancreatic ampulla, pancreas and gastric antrum, and patients with post-operative relapsed cancers, usually have biliary obstructions in addition to gastric outlet obstruction. To those patients with old age, tumor invasion of the large vessels, distant metastasis, complex anatomical structures and intolerability to radical surgery, the palliative treatment modalities with minimal traumatic, fast recovery and satisfactory efficacy should be the priority options [1-6]. With the development of endoscopic techniques as well as the invention of intestinal stents via endoscopic biopsy channel (through the scope, TTS), the gastric outlet stenting becomes easy and convenient [7-9].

Percutaneous transhepatic biliary drainage (PTCD) with stenting in the treatment of obstructive jaundice has been widely used and has become the first choice for the palliative treatment of malignant obstructive jaundice [6-8]. In this study, we attempted to observe the palliative efficacy of the combined intestinal and biliary stenting on the malignant gastric outlet obstruction with common bile duct obstruction.

## Materials and Methods

### Patients

All patients were from the Departments of Gastroenterology and General Surgery in Provincial Hospital Affiliated to Shandong University between January 2001 and September 2007. All patients had malignant gastric outlet obstruction and bile duct obstruction, these patients had one or more of the following, old age, weak condition, cardiopulmonary diseases, tumor invasion of the large vessel, or distant metastasis. These patients could not tolerate or refused radical surgery. Patients with severe bleeding tendency, severe ascites, or any signs intolerable to endoscopy were excluded from this treatment. A total of 32 cases were included in this study, 19 male and 13 female, average age 61.3 years, ranging from 46 to 83 years. These patients included ten cases of duodenal cancer, 6 recurred gastric antrum cancer after operations, 6 periampullary cancer, 8 pancreatic cancer, 2 lower common bile duct cancer. All patients presented with vomiting, inability to eat or other gastrointestinal obstruction symptoms and jaundice, pruritus and other biliary obstruction symptoms, total bilirubin 85 - 613 µmol/L. Preoperatively, all patients were diagnosed to have malignant gastric outlet obstruction with stenosis length between 2 - 7 cm, confirmed by endoscopy and/or upper gastrointestinal diatrizoate contrast. In addition, the ultrasound, computed tomography (CT) and/or magnetic resonance cholangiopancreatography (MRCP) were performed to examine the intra- and extra-hepatic bile duct expansion including the common bile duct obstruction, the length of common bile duct obstruction was 1.5 - 4 cm.

### Materials and instruments

Intestinal stenting instruments: Olympus GIF-IT260 electronic endoscopy with a 3.2 mm in diameter biopsy channel; MTN-intestinal expandable stent (Nanjing Minimal Invasive Medical Technology Co., China) with inner diameter 20 mm; zebra guiding wires with diameter of 0.035 in, length 4 m; 5 F straight catheters or multi-purpose catheters; extra-lubricity wire with diameter of 0.035 in, length of 2.6 m.

Biliary stenting instruments: The biliary micro-needle system; the interior and exterior drainage catheters with diameter of 8.5 F, length of 40 cm (Cook, Bloomington, USA); 6 F catheter sheath; extra-lubricity Paramisgurnus wire; 5 F multi-purpose catheter; dilating balloon with diameter of 0.8 - 1.0 cm, length of 3 - 4 cm; hard extra-lubricity wire; MTN-biliary expandable stent (Nanjing Minimal Invasive Medical Technology Co., China), with diameter 0.8 cm, length 5 - 8 cm.

### Stenting procedures

Preoperative preparations included obtaining of consent from patients. Vitamin K_1_ and calcium gluconate were administered for three days prior to stenting.

During the intestinal stenting, the routine endoscopy procedure was carried out to aspirate the gastric contents and to observe the stenosis, 40 - 60 ml of 76% diatrizoate was injected via the biopsy channel to mark the range of stenosis, based on which the appropriate length of stent was selected. If the endoscopy could not pass the stenosis, a Zebra wire was inserted first, then the stents were placed under the guidance of the wire. After the stent placement, the endoscopy and X-ray were used to examine the location and expansion of the stents; if necessary, adjustment of stent position was performed under endoscopy. If two stents were needed in one patient, the distal one must be placed first, then the proximal one.

During the biliary stent placement, we usually used the right axillary midline between 7 and 8 intercostal as the puncture point when patients were in supine position. After successful puncture, the percutaneous transhepatic cholangiography (PTC) was performed to observe the location and the length of obstruction, after expansion with the 6 F catheter sheath, a 5 F multipurpose catheter was placed at the proximal of the stenosis first, then advanced through the stenosis to reach the intestinal stent along the wire, cholangiography was performed in order to check the location and the extent of biliary stenosis. Afterwards, the wire was replaced with a hard wire, and a dilating balloon was inserted to expand the biliary stenosis and the mesh of the intestinal stent, the biliary stents then were inserted along the wire. After biliary stenting, the interior and exterior drainage catheters with diameter of 8.5 F, length of 40 cm were placed, with the distal end located in the duodenum, the proximal in the right hepatic duct or hepatic duct, and it was connected to the exterior drainage.

After the stents placement, close observations were made to jaundice, vomiting, abdominal pain, abdominal distension, vomiting blood or melena. The liquid to semi-liquid diet was given 24 h after stenting; the volume and features of drainage were recorded. Biliary flushing with 80,000 units of gentamycin in 100 ml of 0.9% NaCl solution was performed daily for three days, then the exterior drainage was closed. If the interior drainage was patent, the exterior drainage catheter was removed. Three to 7 days after the stenting, oral contrast agent imaging was performed to observe the status of the intestinal stent patency. The hematology, liver function, bilirubin, blood chemistry were ananlyzed. If jaundice ameliorated significantly, and blood bilirubin decreased more than 50% of pre-stenting level, the patients could be discharged. Follow-up was made by telephone or clinic visit.

### Stent patency evaluation

Two weeks after stenting, if blood total bilirubin (TBil) decreased ≥ 50% and patient could tolerate semi-liquid diet, it was considered complete response; if TBil decresed less than 50% and patient tolerated only liquid diet, it was partial response; if TBil unchanged and patient could not tolerate liquid diet, it was non response; if TBil elevated and patient could not tolerate liquid diet, and the gastrointestinal decompression was still required, it was deterioration [1-9].

### Statistical analysis

SPSS 13.0 statistical program was used for analyzing the data, data was expressed in Mean ± SD, inter-group comparison used Student’s *t* test, the P value of less than 0.05 was considered significantly different.

## Results

### Effecacy of stenting

**Figure 1:**
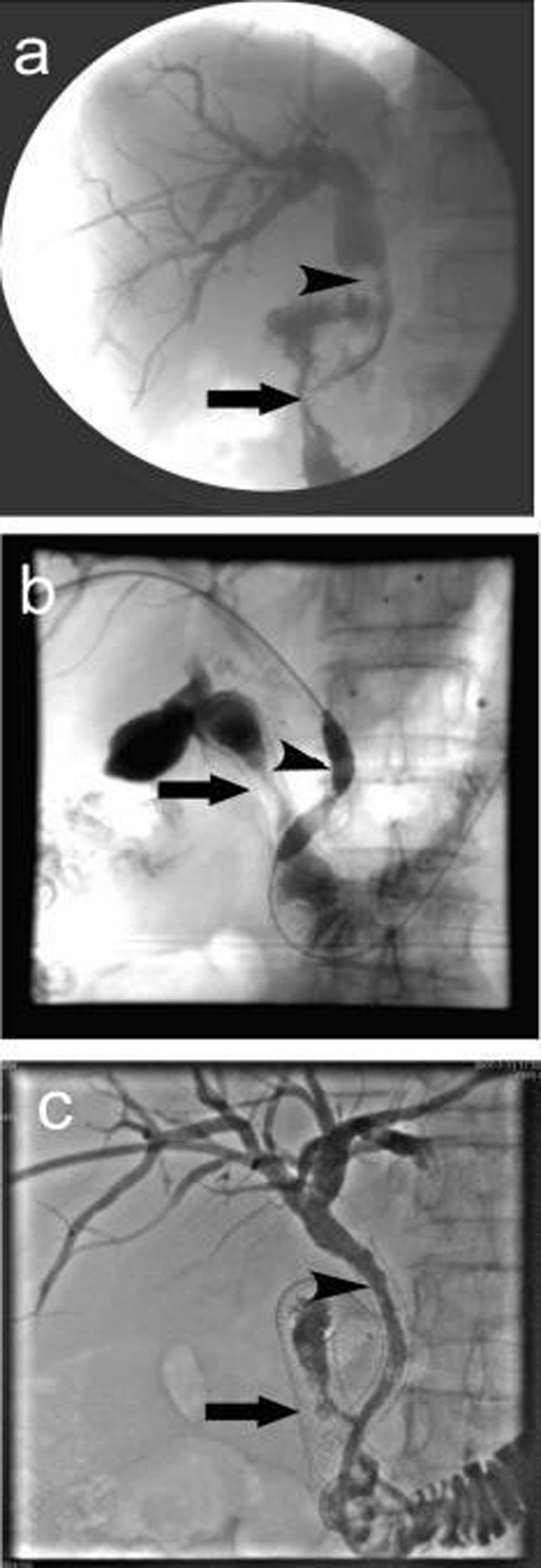
Procedure of double stenting. (a) Duodenal stenosis combined with common bile duct lesions. The arrow indicates descending duodenal stenosis, the arrow head shows the irregular contrast deficiency of lower common bile duct, which might be invaded by tumors which led to the expansion of common bile duct above the stenosis; (b) Biliary stent placement, the arrow shows duodenal stent, the arrow head shows balloon dilatation of common bile duct stenosis and the mesh of intestinal stent; (c) Biliary stent placement, the arrow shows duodenal stent, the arrow head shows biliary stent. The cholangiography showed both were patent.

**Figure 2:**
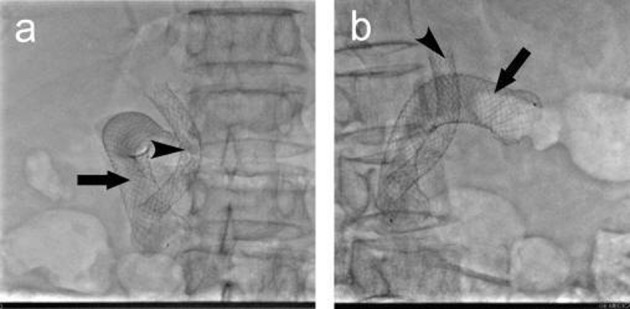
The relationship of the intestinal and biliary stents. (a) anteroposterior; (b) lateral.

A total of 37 intestinal stents were placed in 32 patients, single stent was placed in 27 patients; in 5 patients, due to the long duodenal stenosis, double stents were placed in each patient. A total of 32 biliary stents were placed in 32 patients. The cholangiography 3 days after stenting proved patency of both intestinal and biliary stents, the success rate of combined intestinal and biliary stenting was 100%. Figure 1a shows the extrahepatic obstructive jaundice with biliary infection before stent placement, PTC revealed common bile duct lesions accompanied by duodenal stenosis. Figure 1b shows the biliary stenting following the intestinal stent was placed, PTCD was carried out to place the biliary stent. Figure 1c shows the biliary stent across the mesh of intestinal stent, the biliary and intestinal stents were both revealed patent. Figure 2 shows the relationship between biliary stent and intestinal stent.

### Short-term efficacy

The obstructive symptoms in all the 32 patients improved gradually after stenting, all the patients had complete response or partial response. One week after stenting, the jaundice, itching and other symptoms ameliorated. In 21 patients, the serum TBil and direct bilirubin (DBil) decreased more than 50% one week after stenting; in all the 32 patients, the alanine aminotransferase (ALT), aspartate aminotransferase (AST), alkaline phosphatase ( ALP), γ-glutamyltranspeptidase enzyme (γ-GT) decreased significantly (Table 1).

**Table 1:** Serum bilirubin and liver function before and 2 weeks after stenting

	Pre-stenting	Post-stenting (2 weeks)	P value
N	32	32	
TBil (µmol/L)	276.54 ± 72.67	116.37 ± 48.42	< 0.01
DBil (µmol/L)	152.32 ± 42.64	65.70 ± 31.93	< 0.01
ALT (U/L)	109.22 ± 37.56	46.77 ± 33.92	< 0.01
AST (U/L)	72.38 ± 13.86	34.17 ± 18.20	< 0.01
ALP (U/L)	486.35 ± 129.67	117.56 ± 93.42	< 0.01
γ-GT (U/L)	603.22 ± 145.79	247.83 ± 63.07	< 0.01

### Complications

In some patients, minor hemorrhage occurred around the stent during intestinal stent placement, after flushing with saline and spraying hemostat drug locally, no further bleeding occurred. There was no gastrointestinal perforation observed. All 32 patients had upper abdominal pain, nausea or other discomforts immediately after stenting, these symptoms relieved or disappeared 3 – 5 days after. If the pain was marked without perforation, painkillers could be administered. After biliary stenting, serum amylase elevated transiently in 7 patients. One patient had acute pancreatitis after stenting, and was cured by conservative treatment. Three patients had drainage blockage or infection, these were cured by repeated flushing with antibiotics. Two patients had gastric outlet restenosis in the first month, one was caused by food blockade; another was caused by outgrowth of the tumor through the stent mesh which was relieved by re-endoscopic treatment. There were no biliary leak, abdominal bleeding, drainage catheter migration, stents migration and puncture tunnel bleeding.

### Survival

Twenty eight patients were followed up, 2 patients died within 2 months after stenting; 5 died 3-4 months after stenting; 3 died 5-6 months after stenting. The longest survival was 11 months, the average survival time was 164 days.

## Discussion

Patients with malignant gastric outlet obstruction combining with bile duct obstruction may undergo palliative surgeries, such as choledochojejunostomy, gastrojejunostomy, these modalities are traumatic and have complications, therefore, they are now gradually replaced by less traumatic methods such as ERCP, PTCD, intestinal stenting [10-17]. However, some patients with lesions of antral and/or duodenal stenosis or tumor invasion of the papilla are impossible to be performed ERCP, so the PTCD plus metal expandable stent implantation could overcome these shortcomings [5-7]. The only common bile duct drainage is not effective either because of duodenum stenosis which always leads to poor drainage or because of bile reflux to the stomach that would not achieve physiological effects. In such patients, due to the biliary and gastric outlet obstruction occur simultaneously or one after another, the first is to clear the intestinal tract, then to place the intestinal tract stent, the biliary stenting can be performed at the same time as or after the intestinal stenting. If intestinal stent is needed because of gastric outlet obstruction after biliary stent placement, though the two stents may overlap or cover the papilla opening, due to the large mesh of intestinal stent, it will not affect the biliary stent drainage [1, 2, 8, 9, 11, 18].

Intestinal self-expandable metal stent implantation is one of the effective ways to treat malignant gastric outlet obstruction [9, 11, 16, 19]. Due to the long route from the mouth to gastric outlet, the stent placement is difficult. In1996, Feretis et al adopted an extended catheter to prevent the delivery system from rolling in the stomach. In 1997, Scott Mackie adopted the stiff wire and a special delivery system which greatly improved the success of stent placement [5, 6, 10, 15]. In 2003, the invention of TTS intestinal stent makes stenting easier and time saving, which is gradually accepted in clinical work [7-9].

In this study, by combining fluoroscopy with endoscopy, the intestinal stenting was all successful in the first attempt. No bleeding, perforation, or other complications were observed, indicating that this method is safe and efficacious. The adequate preoperative preparation is an important factor for the success of stening, such as gastrointestinal decompression, gastric lavage and nutrition support. The injection of the contrast agent through the endoscopic biopsy channel to mark the stenosis is important for choosing appropriate stent. If the endodcopy could go cross the stenosis, the stent could be released under the surveillance of endoscopy; if not, we advanced the catheter and extra lubricity wire first, then X-ray was used to measure the range of the stenosis and to mark the both ends. The catheter must be guided by the wire and advanced to the ends as far as possible, then the hard extra lubricity wire was used in the upper jejunum in order to place the stent successfully, because the hard extra lubricity wire can not fold in the gastrointestinal tract [20-25].

The PTCD with stenting placement is a frequently used method for bilary reconstruction and alleviation of jaundice. In 1974, PTCD was firstly used by Molnar and Stocknm, in the late 1980s, the stent placement started in some countries. Due to the large mesh and unique weaving of the intestinal stents, the biliary stents can cross it. For the lower common bile duct obstruction, the PTCD plus stenting is simple and convenient, especially for those patients with intestinal obstruction. With the ongoing development of instruments and improved PTCD technology, its successful rate is currently close to 100% [10-12]. In intestinal obstruction, for restoration of physiological functions, the adequate interior and exterior biliary drainage are necessary. The PTCD plus stenting should be recommended for alleviating jaundice in cancer patients with gastrointestinal tract reconstruction [1, 2, 8, 19, 20, 26].

The use of the grid-type stents makes it easier to place the two stents at the same time, this avoids the suppression of the pancreatic duct openings which may result in pancreatic obstructions and pancreatitis. The patients usually feel painful when the intestinal stent mesh is being expanded, when this occur, the painkillers might be administered. The biliary stent should not exceed half of the intestinal stent lumen in order not to affect the patency of the intestinal stent. The indications of stenting placement are very important, these include: (1) the preoperative ultrasound, CT, or MRI confirmed distant metastasis and, radical surgery is impossible; (2) the patients general condition is severely deteriorated, or with severe heart or lung diseases, laparotomy surgery is impossible; (3) patients or their families refuse open surgery; (4) the malignant recurrence surrounding the anastomosis after operation, and the adjacent organs are invaded. The contraindications of the combined stent placement include massive ascites; multiple obstructions of the intestinal tract; and severe bleeding tendency.

In the patients with malignant obstructive jaundice, if no effective biliary drainage is carried out, the survival time is usually around 70 days, and eventually the patients die of liver failure [11, 21, 25]. In this study, the survival time we observed was 164 days in average, which was significantly longer than those without biliary drainage in others’ reports. In our study, we observed a small number of patients, the overall efficacy need further evaluated.

We used the palliative approach to reconstruct biliary duct, so the short-term effect was obvious, but with the tumor growth, invasion and suppression to adjacent organs, the quality of patient life and the survival time will be affected over time. Hence, the combined chemotherapy, radiotherapy and endoscopic therapy should be considered to prolong survival.

In short, the combined intestinal and biliary Stenting is a safe, minimal traumatic, effective treatment for malignant gastric outlet obstruction with common bile duct obstruction, with less complications, it is useful to improve the patient’s life quality and survival time.
